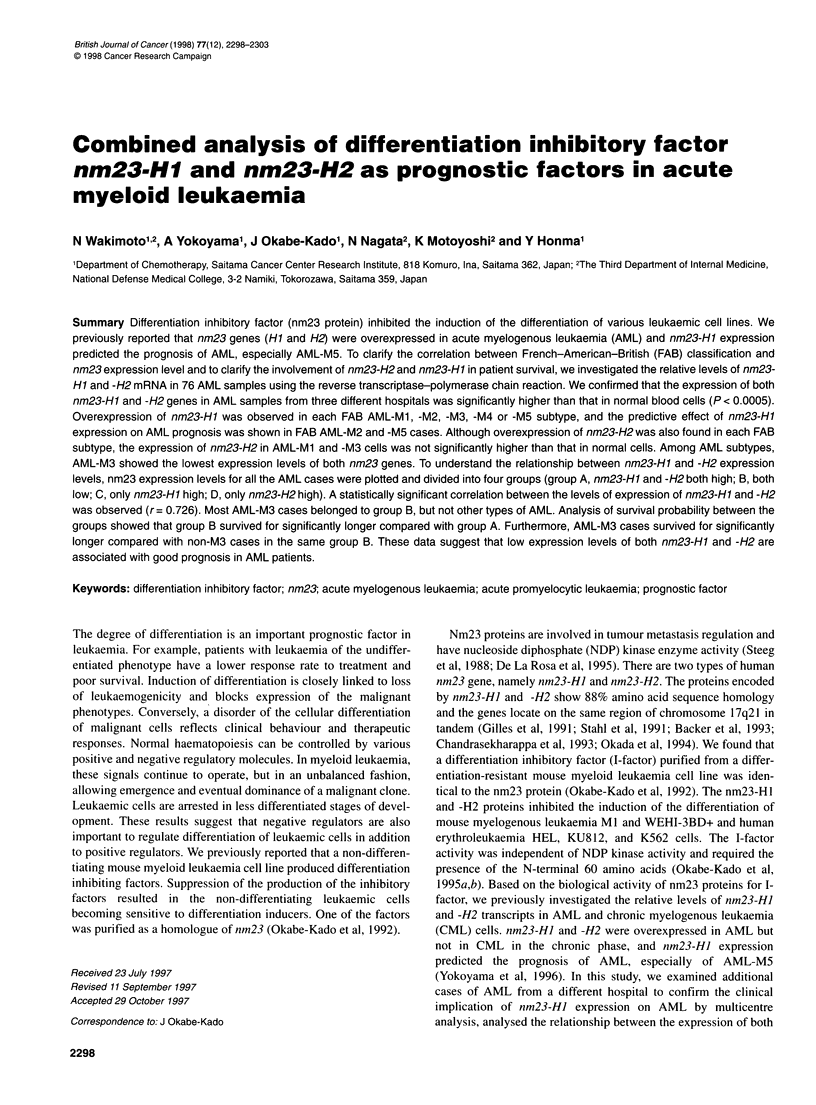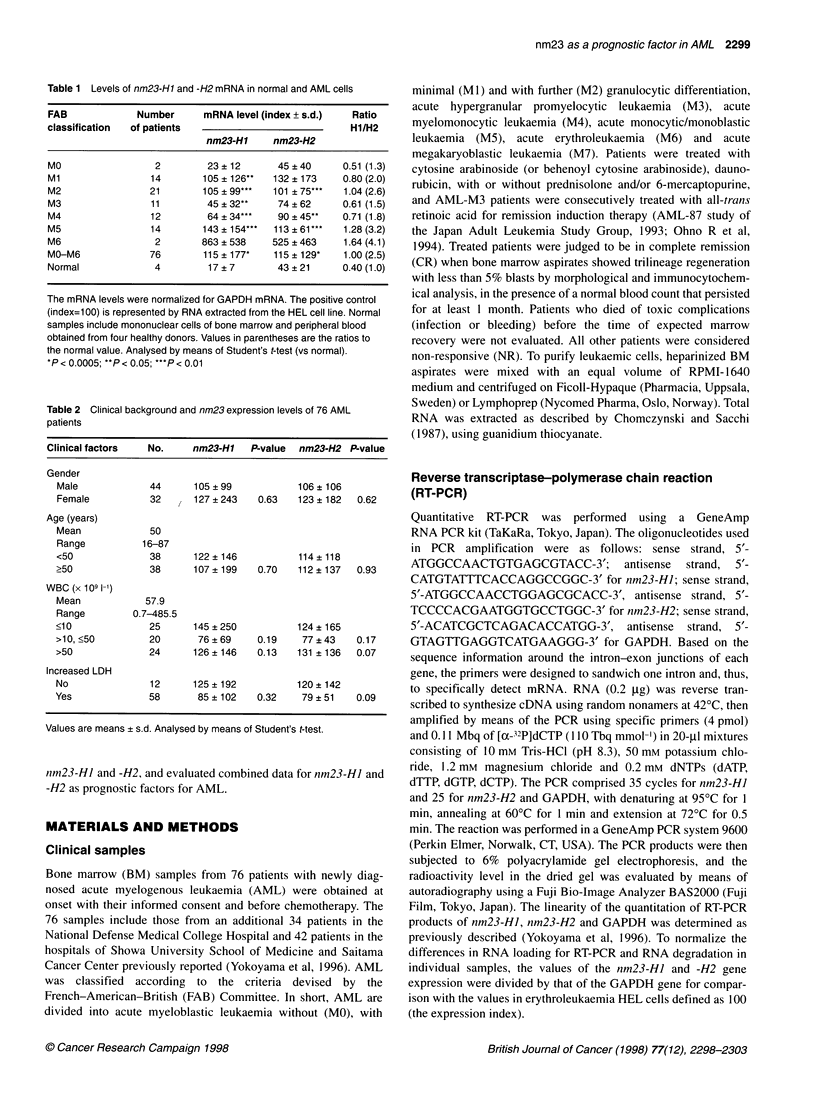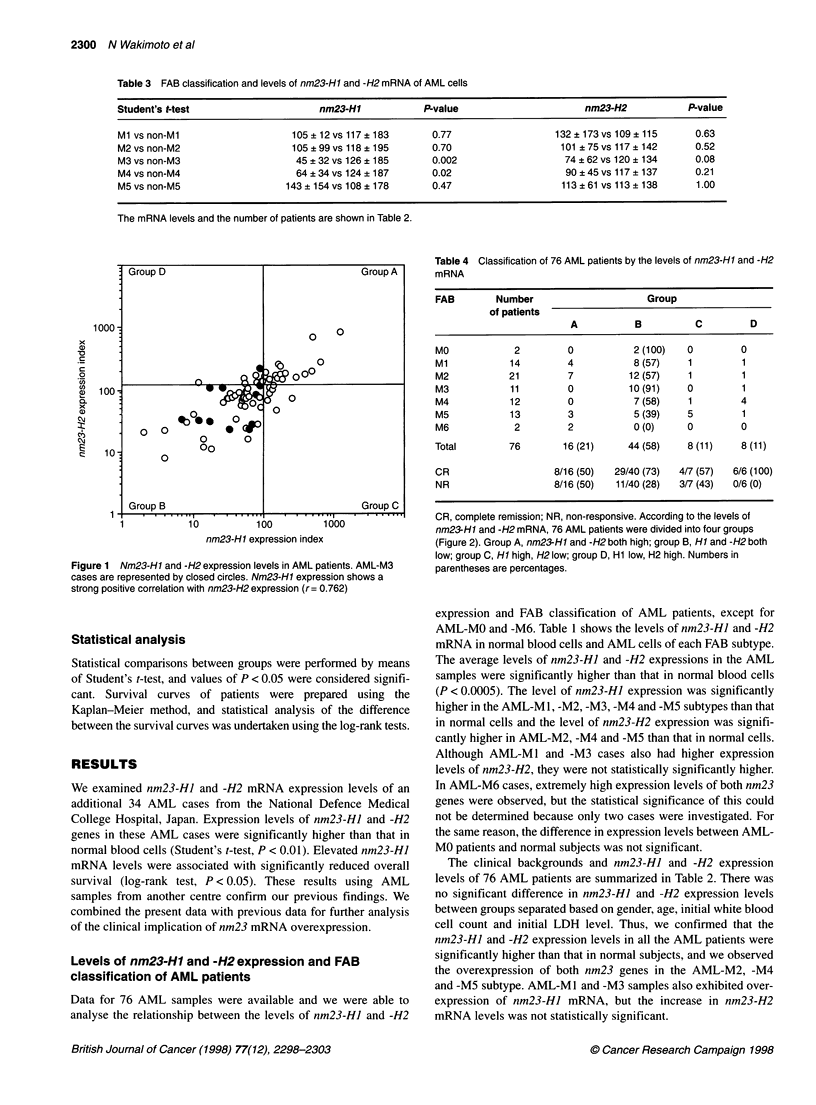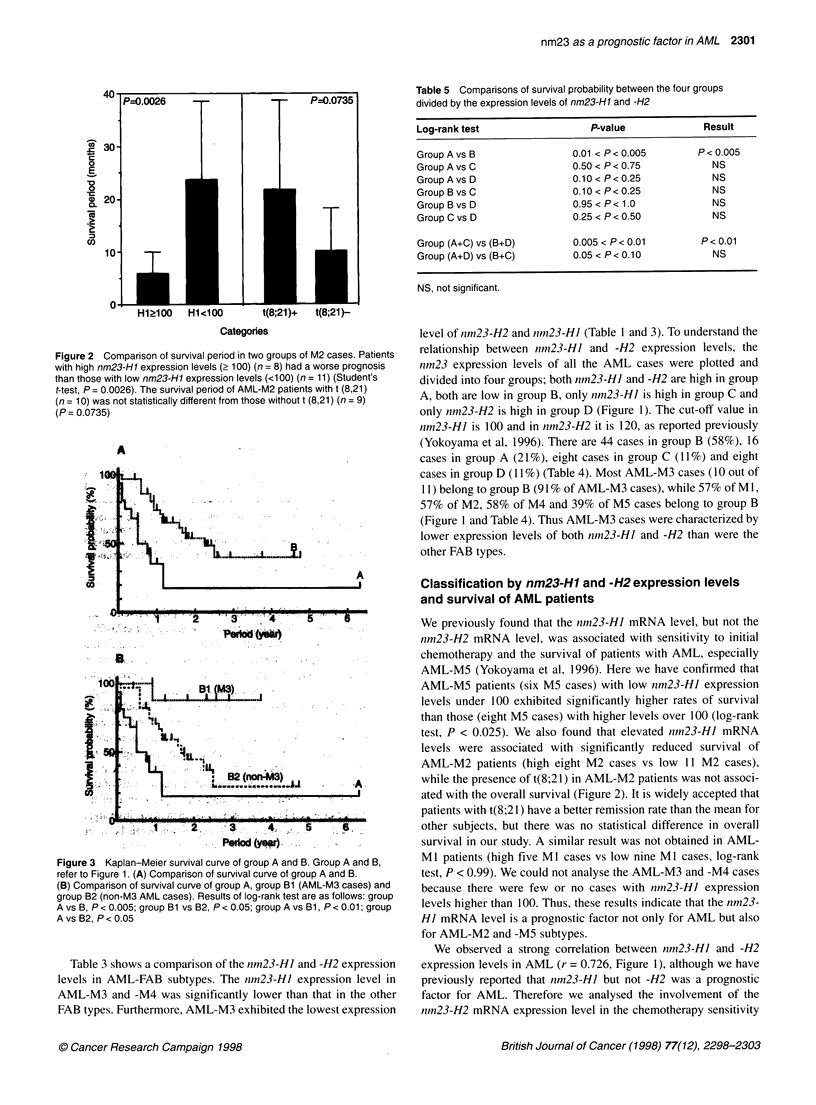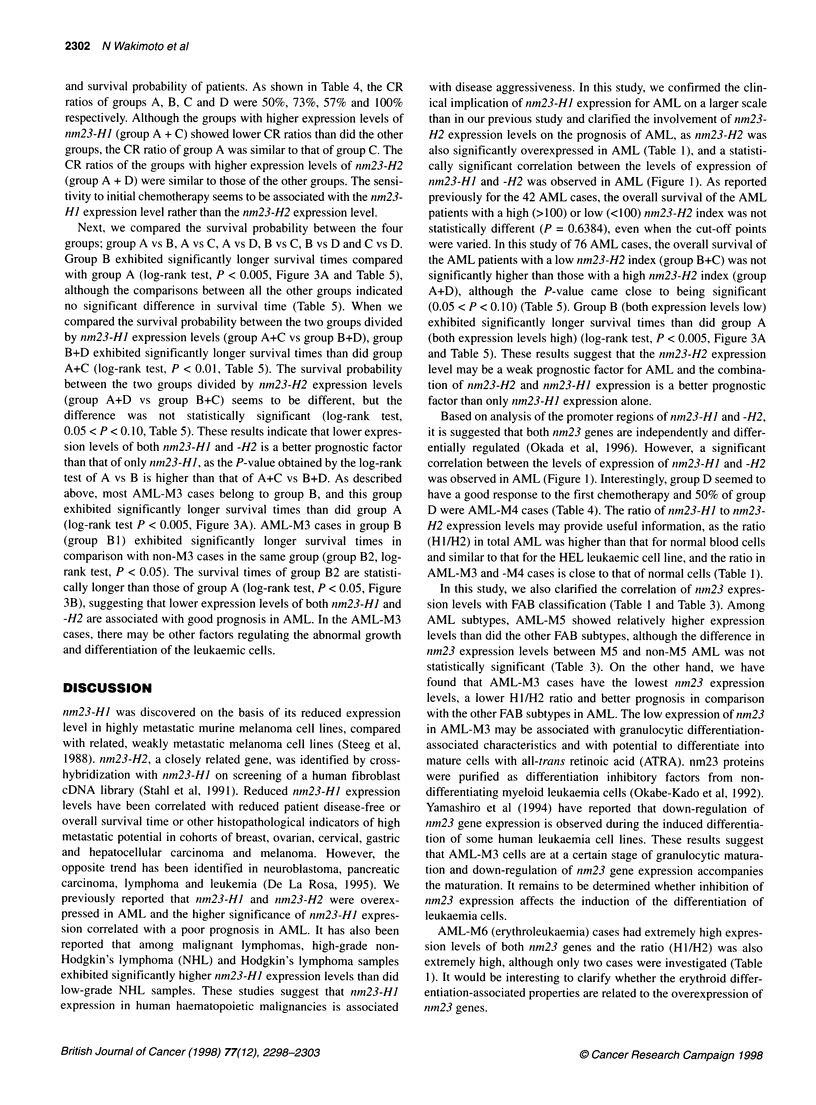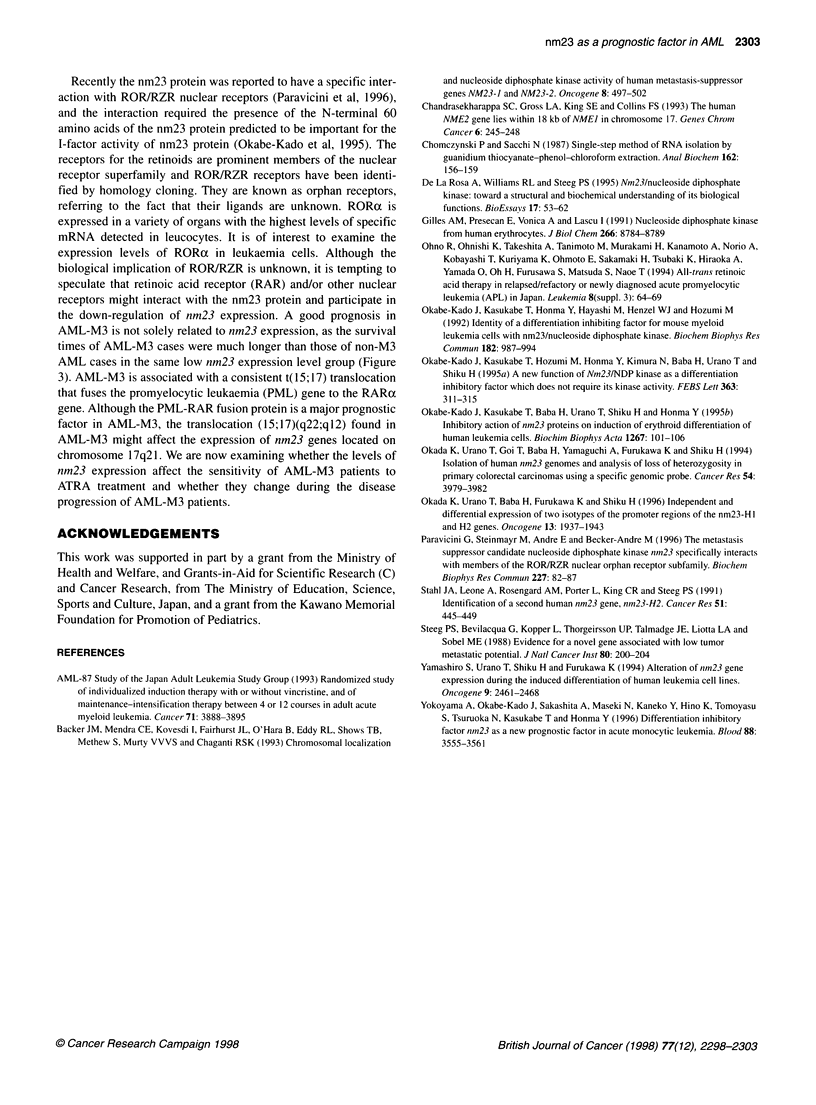# Combined analysis of differentiation inhibitory factor nm23-H1 and nm23-H2 as prognostic factors in acute myeloid leukaemia.

**DOI:** 10.1038/bjc.1998.382

**Published:** 1998-06

**Authors:** N. Wakimoto, A. Yokoyama, J. Okabe-Kado, N. Nagata, K. Motoyoshi, Y. Honma

**Affiliations:** Department of Chemotherapy, Saitama Cancer Center Research Institute, Ina, Japan.

## Abstract

Differentiation inhibitory factor (nm23 protein) inhibited the induction of the differentiation of various leukaemic cell lines. We previously reported that nm23 genes (H1 and H2) were overexpressed in acute myelogenous leukaemia (AML) and nm23-H1 expression predicted the prognosis of AML, especially AML-M5. To clarify the correlation between French-American-British (FAB) classification and nm23 expression level and to clarify the involvement of nm23-H2 and nm23-H1 in patient survival, we investigated the relative levels of nm23-H1 and -H2 mRNA in 76 AML samples using the reverse transcriptase-polymerase chain reaction. We confirmed that the expression of both nm23-H1 and -H2 genes in AML samples from three different hospitals was significantly higher than that in normal blood cells (P < 0.0005). Overexpression of nm23-H1 was observed in each FAB AML-M1, -M2, -M3, -M4 or -M5 subtype, and the predictive effect of nm23-H1 expression on AML prognosis was shown in FAB AML-M2 and -M5 cases. Although overexpression of nm23-H2 was also found in each FAB subtype, the expression of nm23-H2 in AML-M1 and -M3 cells was not significantly higher than that in normal cells. Among AML subtypes, AML-M3 showed the lowest expression levels of both nm23 genes. To understand the relationship between nm23-H1 and -H2 expression levels, nm23 expression levels for all the AML cases were plotted and divided into four groups (group A, nm23-H1 and -H2 both high; B, both low; C, only nm23-H1 high; D, only nm23-H2 high). A statistically significant correlation between the levels of expression of nm23-H1 and -H2 was observed (r= 0.726). Most AML-M3 cases belonged to group B, but not other types of AML. Analysis of survival probability between the groups showed that group B survived for significantly longer compared with group A. Furthermore, AML-M3 cases survived for significantly longer compared with non-M3 cases in the same group B. These data suggest that low expression levels of both nm23-H1 and -H2 are associated with good prognosis in AML patients.


British Journal of Cancer (1998) 77(12), 2298–2303
© 1998 Cancer Research Campaign

### Combined analysis of differentiation inhibitory factor *nm23-H1* and *nm23-H2* as prognostic factors in acute myeloid leukaemia

**N Wakimoto[1,2], A Yokoyama[1], J Okabe-Kado[1], N Nagata[2], K Motoyoshi[2] and Y Honma[1]**

[1]Department of Chemotherapy, Saitama Cancer Center Research Institute, 818 Komuro, Ina, Saitama 362, Japan; [2]The Third Department of Internal Medicine, National Defense Medical College, 3-2 Namiki, Tokorozawa, Saitama 359, Japan

**Summary** Differentiation inhibitory factor (nm23 protein) inhibited the induction of the differentiation of various leukaemic cell lines. We previously reported that *nm23* genes (*H1* and *H2*) were overexpressed in acute myelogenous leukaemia (AML) and *nm23-H1* expression predicted the prognosis of AML, especially AML-M5. To clarify the correlation between French–American–British (FAB) classification and *nm23* expression level and to clarify the involvement of *nm23-H2* and *nm23-H1* in patient survival, we investigated the relative levels of *nm23-H1* and *-H2* mRNA in 76 AML samples using the reverse transcriptase–polymerase chain reaction. We confirmed that the expression of both *nm23-H1* and *-H2* genes in AML samples from three different hospitals was significantly higher than that in normal blood cells ($P < 0.0005$). Overexpression of *nm23-H1* was observed in each FAB AML-M1, -M2, -M3, -M4 or -M5 subtype, and the predictive effect of *nm23-H1* expression on AML prognosis was shown in FAB AML-M2 and -M5 cases. Although overexpression of *nm23-H2* was also found in each FAB subtype, the expression of *nm23-H2* in AML-M1 and -M3 cells was not significantly higher than that in normal cells. Among AML subtypes, AML-M3 showed the lowest expression levels of both *nm23* genes. To understand the relationship between *nm23-H1* and *-H2* expression levels, nm23 expression levels for all the AML cases were plotted and divided into four groups (group A, *nm23-H1* and *-H2* both high; B, both low; C, only *nm23-H1* high; D, only *nm23-H2* high). A statistically significant correlation between the levels of expression of *nm23-H1* and *-H2* was observed ($r = 0.726$). Most AML-M3 cases belonged to group B, but not other types of AML. Analysis of survival probability between the groups showed that group B survived for significantly longer compared with group A. Furthermore, AML-M3 cases survived for significantly longer compared with non-M3 cases in the same group B. These data suggest that low expression levels of both *nm23-H1* and *-H2* are associated with good prognosis in AML patients.

**Keywords:** differentiation inhibitory factor; *nm23*; acute myelogenous leukaemia; acute promyelocytic leukaemia; prognostic factor

The degree of differentiation is an important prognostic factor in leukaemia. For example, patients with leukaemia of the undifferentiated phenotype have a lower response rate to treatment and poor survival. Induction of differentiation is closely linked to loss of leukaemogenicity and blocks expression of the malignant phenotypes. Conversely, a disorder of the cellular differentiation of malignant cells reflects clinical behaviour and therapeutic responses. Normal haematopoiesis can be controlled by various positive and negative regulatory molecules. In myeloid leukaemia, these signals continue to operate, but in an unbalanced fashion, allowing emergence and eventual dominance of a malignant clone. Leukaemic cells are arrested in less differentiated stages of development. These results suggest that negative regulators are also important to regulate differentiation of leukaemic cells in addition to positive regulators. We previously reported that a non-differentiating mouse myeloid leukaemia cell line produced differentiation inhibiting factors. Suppression of the production of the inhibitory factors resulted in the non-differentiating leukaemic cells becoming sensitive to differentiation inducers. One of the factors was purified as a homologue of *nm23* (Okabe-Kado et al, 1992).

*Received 23 July 1997*
*Revised 11 September 1997*
*Accepted 29 October 1997*

*Correspondence to:* J Okabe-Kado

Nm23 proteins are involved in tumour metastasis regulation and have nucleoside diphosphate (NDP) kinase enzyme activity (Steeg et al, 1988; De La Rosa et al, 1995). There are two types of human *nm23* gene, namely *nm23-H1* and *nm23-H2*. The proteins encoded by *nm23-H1* and *-H2* show 88% amino acid sequence homology and the genes locate on the same region of chromosome 17q21 in tandem (Gilles et al, 1991; Stahl et al, 1991; Backer et al, 1993; Chandrasekharappa et al, 1993; Okada et al, 1994). We found that a differentiation inhibitory factor (I-factor) purified from a differentiation-resistant mouse myeloid leukaemia cell line was identical to the nm23 protein (Okabe-Kado et al, 1992). The nm23-H1 and -H2 proteins inhibited the induction of the differentiation of mouse myelogenous leukaemia M1 and WEHI-3BD+ and human erythroleukaemia HEL, KU812, and K562 cells. The I-factor activity was independent of NDP kinase activity and required the presence of the N-terminal 60 amino acids (Okabe-Kado et al, 1995*a*,*b*). Based on the biological activity of nm23 proteins for I-factor, we previously investigated the relative levels of *nm23-H1* and *-H2* transcripts in AML and chronic myelogenous leukaemia (CML) cells. *nm23-H1* and *-H2* were overexpressed in AML but not in CML in the chronic phase, and *nm23-H1* expression predicted the prognosis of AML, especially of AML-M5 (Yokoyama et al, 1996). In this study, we examined additional cases of AML from a different hospital to confirm the clinical implication of *nm23-H1* expression on AML by multicentre analysis, analysed the relationship between the expression of both

**Table 1** Levels of *nm23-H1* and *-H2* mRNA in normal and AML cells

| FAB classification | Number of patients | mRNA level (index ± s.d.) | | Ratio H1/H2 |
|---|---|---|---|---|
| | | *nm23-H1* | *nm23-H2* | |
| M0 | 2 | 23 ± 12 | 45 ± 40 | 0.51 (1.3) |
| M1 | 14 | 105 ± 126** | 132 ± 173 | 0.80 (2.0) |
| M2 | 21 | 105 ± 99*** | 101 ± 75*** | 1.04 (2.6) |
| M3 | 11 | 45 ± 32** | 74 ± 62 | 0.61 (1.5) |
| M4 | 12 | 64 ± 34*** | 90 ± 45** | 0.71 (1.8) |
| M5 | 14 | 143 ± 154*** | 113 ± 61*** | 1.28 (3.2) |
| M6 | 2 | 863 ± 538 | 525 ± 463 | 1.64 (4.1) |
| M0–M6 | 76 | 115 ± 177* | 115 ± 129* | 1.00 (2.5) |
| Normal | 4 | 17 ± 7 | 43 ± 21 | 0.40 (1.0) |

The mRNA levels were normalized for GAPDH mRNA. The positive control (index=100) is represented by RNA extracted from the HEL cell line. Normal samples include mononuclear cells of bone marrow and peripheral blood obtained from four healthy donors. Values in parentheses are the ratios to the normal value. Analysed by means of Student's *t*-test (vs normal). *$P < 0.0005$; **$P < 0.05$; ***$P < 0.01$

**Table 2** Clinical background and *nm23* expression levels of 76 AML patients

| Clinical factors | No. | *nm23-H1* | *P*-value | *nm23-H2* | *P*-value |
|---|---|---|---|---|---|
| Gender | | | | | |
| Male | 44 | 105 ± 99 | | 106 ± 106 | |
| Female | 32 | 127 ± 243 | 0.63 | 123 ± 182 | 0.62 |
| Age (years) | | | | | |
| Mean | 50 | | | | |
| Range | 16–87 | | | | |
| <50 | 38 | 122 ± 146 | | 114 ± 118 | |
| ≥50 | 38 | 107 ± 199 | 0.70 | 112 ± 137 | 0.93 |
| WBC ($\times 10^9$ $l^{-1}$) | | | | | |
| Mean | 57.9 | | | | |
| Range | 0.7–485.5 | | | | |
| ≤10 | 25 | 145 ± 250 | | 124 ± 165 | |
| >10, ≤50 | 20 | 76 ± 69 | 0.19 | 77 ± 43 | 0.17 |
| >50 | 24 | 126 ± 146 | 0.13 | 131 ± 136 | 0.07 |
| Increased LDH | | | | | |
| No | 12 | 125 ± 192 | | 120 ± 142 | |
| Yes | 58 | 85 ± 102 | 0.32 | 79 ± 51 | 0.09 |

Values are means ± s.d. Analysed by means of Student's *t*-test.

*nm23-H1* and *-H2*, and evaluated combined data for *nm23-H1* and *-H2* as prognostic factors for AML.

### MATERIALS AND METHODS

### Clinical samples

Bone marrow (BM) samples from 76 patients with newly diagnosed acute myelogenous leukaemia (AML) were obtained at onset with their informed consent and before chemotherapy. The 76 samples include those from an additional 34 patients in the National Defense Medical College Hospital and 42 patients in the hospitals of Showa University School of Medicine and Saitama Cancer Center previously reported (Yokoyama et al, 1996). AML was classified according to the criteria devised by the French–American–British (FAB) Committee. In short, AML are divided into acute myeloblastic leukaemia without (M0), with minimal (M1) and with further (M2) granulocytic differentiation, acute hypergranular promyelocytic leukaemia (M3), acute myelomonocytic leukaemia (M4), acute monocytic/monoblastic leukaemia (M5), acute erythroleukaemia (M6) and acute megakaryoblastic leukaemia (M7). Patients were treated with cytosine arabinoside (or behenoyl cytosine arabinoside), daunorubicin, with or without prednisolone and/or 6-mercaptopurine, and AML-M3 patients were consecutively treated with all-*trans* retinoic acid for remission induction therapy (AML-87 study of the Japan Adult Leukemia Study Group, 1993; Ohno R et al, 1994). Treated patients were judged to be in complete remission (CR) when bone marrow aspirates showed trilineage regeneration with less than 5% blasts by morphological and immunocytochemical analysis, in the presence of a normal blood count that persisted for at least 1 month. Patients who died of toxic complications (infection or bleeding) before the time of expected marrow recovery were not evaluated. All other patients were considered non-responsive (NR). To purify leukaemic cells, heparinized BM aspirates were mixed with an equal volume of RPMI-1640 medium and centrifuged on Ficoll-Hypaque (Pharmacia, Uppsala, Sweden) or Lymphoprep (Nycomed Pharma, Oslo, Norway). Total RNA was extracted as described by Chomczynski and Sacchi (1987), using guanidium thiocyanate.

### Reverse transcriptase–polymerase chain reaction (RT-PCR)

Quantitative RT-PCR was performed using a GeneAmp RNA PCR kit (TaKaRa, Tokyo, Japan). The oligonucleotides used in PCR amplification were as follows: sense strand, 5′-ATGGCCAACTGTGAGCGTACC-3′; antisense strand, 5′-CATGTATTTCACCAGGCCGGC-3′ for *nm23-H1*; sense strand, 5′-ATGGCCAACCTGGAGCGCACC-3′, antisense strand, 5′-TCCCCACGAATGGTGCCTGGC-3′ for *nm23-H2*; sense strand, 5′-ACATCGCTCAGACACCATGG-3′, antisense strand, 5′-GTAGTTGAGGTCATGAAGGG-3′ for GAPDH. Based on the sequence information around the intron–exon junctions of each gene, the primers were designed to sandwich one intron and, thus, to specifically detect mRNA. RNA (0.2 μg) was reverse transcribed to synthesize cDNA using random nonamers at 42°C, then amplified by means of the PCR using specific primers (4 pmol) and 0.11 Mbq of [$\alpha$-$^{32}$P]dCTP (110 Tbq $mmol^{-1}$) in 20-μl mixtures consisting of 10 mM Tris-HCl (pH 8.3), 50 mM potassium chloride, 1.2 mM magnesium chloride and 0.2 mM dNTPs (dATP, dTTP, dGTP, dCTP). The PCR comprised 35 cycles for *nm23-H1* and 25 for *nm23-H2* and GAPDH, with denaturing at 95°C for 1 min, annealing at 60°C for 1 min and extension at 72°C for 0.5 min. The reaction was performed in a GeneAmp PCR system 9600 (Perkin Elmer, Norwalk, CT, USA). The PCR products were then subjected to 6% polyacrylamide gel electrophoresis, and the radioactivity level in the dried gel was evaluated by means of autoradiography using a Fuji Bio-Image Analyzer BAS2000 (Fuji Film, Tokyo, Japan). The linearity of the quantitation of RT-PCR products of *nm23-H1*, *nm23-H2* and GAPDH was determined as previously described (Yokoyama et al, 1996). To normalize the differences in RNA loading for RT-PCR and RNA degradation in individual samples, the values of the *nm23-H1* and *-H2* gene expression were divided by that of the GAPDH gene for comparison with the values in erythroleukaemia HEL cells defined as 100 (the expression index).

© Cancer Research Campaign 1998

**Table 3** FAB classification and levels of *nm23-H1* and *-H2* mRNA of AML cells

| Student's *t*-test | *nm23-H1* | *P*-value | *nm23-H2* | *P*-value |
|---|---|---|---|---|
| M1 vs non-M1 | 105 ± 12 vs 117 ± 183 | 0.77 | 132 ± 173 vs 109 ± 115 | 0.63 |
| M2 vs non-M2 | 105 ± 99 vs 118 ± 195 | 0.70 | 101 ± 75 vs 117 ± 142 | 0.52 |
| M3 vs non-M3 | 45 ± 32 vs 126 ± 185 | 0.002 | 74 ± 62 vs 120 ± 134 | 0.08 |
| M4 vs non-M4 | 64 ± 34 vs 124 ± 187 | 0.02 | 90 ± 45 vs 117 ± 137 | 0.21 |
| M5 vs non-M5 | 143 ± 154 vs 108 ± 178 | 0.47 | 113 ± 61 vs 113 ± 138 | 1.00 |

The mRNA levels and the number of patients are shown in Table 2.

**Figure 1** *Nm23-H1* and *-H2* expression levels in AML patients. AML-M3 cases are represented by closed circles. *Nm23-H1* expression shows a strong positive correlation with *nm23-H2* expression ($r = 0.762$)

**Table 4** Classification of 76 AML patients by the levels of *nm23-H1* and *-H2* mRNA

| FAB | Number of patients | Group | | | |
|---|---|---|---|---|---|
| | | A | B | C | D |
| M0 | 2 | 0 | 2 (100) | 0 | 0 |
| M1 | 14 | 4 | 8 (57) | 1 | 1 |
| M2 | 21 | 7 | 12 (57) | 1 | 1 |
| M3 | 11 | 0 | 10 (91) | 0 | 1 |
| M4 | 12 | 0 | 7 (58) | 1 | 4 |
| M5 | 13 | 3 | 5 (39) | 5 | 1 |
| M6 | 2 | 2 | 0 (0) | 0 | 0 |
| Total | 76 | 16 (21) | 44 (58) | 8 (11) | 8 (11) |
| CR | | 8/16 (50) | 29/40 (73) | 4/7 (57) | 6/6 (100) |
| NR | | 8/16 (50) | 11/40 (28) | 3/7 (43) | 0/6 (0) |

CR, complete remission; NR, non-responsive. According to the levels of *nm23-H1* and *-H2* mRNA, 76 AML patients were divided into four groups (Figure 2). Group A, *nm23-H1* and *-H2* both high; group B, *H1* and *-H2* both low; group C, *H1* high, *H2* low; group D, H1 low, H2 high. Numbers in parentheses are percentages.

### Statistical analysis

Statistical comparisons between groups were performed by means of Student's *t*-test, and values of $P < 0.05$ were considered significant. Survival curves of patients were prepared using the Kaplan–Meier method, and statistical analysis of the difference between the survival curves was undertaken using the log-rank tests.

### RESULTS

We examined *nm23-H1* and *-H2* mRNA expression levels of an additional 34 AML cases from the National Defence Medical College Hospital, Japan. Expression levels of *nm23-H1* and *-H2* genes in these AML cases were significantly higher than that in normal blood cells (Student's *t*-test, $P < 0.01$). Elevated *nm23-H1* mRNA levels were associated with significantly reduced overall survival (log-rank test, $P < 0.05$). These results using AML samples from another centre confirm our previous findings. We combined the present data with previous data for further analysis of the clinical implication of *nm23* mRNA overexpression.

### Levels of *nm23-H1* and *-H2* expression and FAB classification of AML patients

Data for 76 AML samples were available and we were able to analyse the relationship between the levels of *nm23-H1* and *-H2* expression and FAB classification of AML patients, except for AML-M0 and -M6. Table 1 shows the levels of *nm23-H1* and *-H2* mRNA in normal blood cells and AML cells of each FAB subtype. The average levels of *nm23-H1* and *-H2* expressions in the AML samples were significantly higher than that in normal blood cells ($P < 0.0005$). The level of *nm23-H1* expression was significantly higher in the AML-M1, -M2, -M3, -M4 and -M5 subtypes than that in normal cells and the level of *nm23-H2* expression was significantly higher in AML-M2, -M4 and -M5 than that in normal cells. Although AML-M1 and -M3 cases also had higher expression levels of *nm23-H2*, they were not statistically significantly higher. In AML-M6 cases, extremely high expression levels of both *nm23* genes were observed, but the statistical significance of this could not be determined because only two cases were investigated. For the same reason, the difference in expression levels between AML-M0 patients and normal subjects was not significant.

The clinical backgrounds and *nm23-H1* and *-H2* expression levels of 76 AML patients are summarized in Table 2. There was no significant difference in *nm23-H1* and *-H2* expression levels between groups separated based on gender, age, initial white blood cell count and initial LDH level. Thus, we confirmed that the *nm23-H1* and *-H2* expression levels in all the AML patients were significantly higher than that in normal subjects, and we observed the overexpression of both *nm23* genes in the AML-M2, -M4 and -M5 subtype. AML-M1 and -M3 samples also exhibited overexpression of *nm23-H1* mRNA, but the increase in *nm23-H2* mRNA levels was not statistically significant.

© Cancer Research Campaign 1998

**Figure 2** Comparison of survival period in two groups of M2 cases. Patients with high *nm23-H1* expression levels (≥ 100) (*n* = 8) had a worse prognosis than those with low *nm23-H1* expression levels (<100) (*n* = 11) (Student's *t*-test, *P* = 0.0026). The survival period of AML-M2 patients with t (8,21) (*n* = 10) was not statistically different from those without t (8,21) (*n* = 9) (*P* = 0.0735)

**Figure 3** Kaplan–Meier survival curve of group A and B. Group A and B, refer to Figure 1. (**A**) Comparison of survival curve of group A and B. (**B**) Comparison of survival curve of group A, group B1 (AML-M3 cases) and group B2 (non-M3 AML cases). Results of log-rank test are as follows: group A vs B, *P* < 0.005; group B1 vs B2, *P* < 0.05; group A vs B1, *P* < 0.01; group A vs B2, *P* < 0.05

Table 3 shows a comparison of the *nm23-H1* and *-H2* expression levels in AML-FAB subtypes. The *nm23-H1* expression level in AML-M3 and -M4 was significantly lower than that in the other FAB types. Furthermore, AML-M3 exhibited the lowest expression level of *nm23-H2* and *nm23-H1* (Table 1 and 3). To understand the relationship between *nm23-H1* and *-H2* expression levels, the *nm23* expression levels of all the AML cases were plotted and divided into four groups; both *nm23-H1* and *-H2* are high in group A, both are low in group B, only *nm23-H1* is high in group C and only *nm23-H2* is high in group D (Figure 1). The cut-off value in *nm23-H1* is 100 and in *nm23-H2* it is 120, as reported previously (Yokoyama et al, 1996). There are 44 cases in group B (58%), 16 cases in group A (21%), eight cases in group C (11%) and eight cases in group D (11%) (Table 4). Most AML-M3 cases (10 out of 11) belong to group B (91% of AML-M3 cases), while 57% of M1, 57% of M2, 58% of M4 and 39% of M5 cases belong to group B (Figure 1 and Table 4). Thus AML-M3 cases were characterized by lower expression levels of both *nm23-H1* and *-H2* than were the other FAB types.

**Table 5** Comparisons of survival probability between the four groups divided by the expression levels of *nm23-H1* and *-H2*

| Log-rank test | *P*-value | Result |
|---|---|---|
| Group A vs B | 0.01 < *P* < 0.005 | *P* < 0.005 |
| Group A vs C | 0.50 < *P* < 0.75 | NS |
| Group A vs D | 0.10 < *P* < 0.25 | NS |
| Group B vs C | 0.10 < *P* < 0.25 | NS |
| Group B vs D | 0.95 < *P* < 1.0 | NS |
| Group C vs D | 0.25 < *P* < 0.50 | NS |
| Group (A+C) vs (B+D) | 0.005 < *P* < 0.01 | *P* < 0.01 |
| Group (A+D) vs (B+C) | 0.05 < *P* < 0.10 | NS |

NS, not significant.

### Classification by *nm23-H1* and *-H2* expression levels and survival of AML patients

We previously found that the *nm23-H1* mRNA level, but not the *nm23-H2* mRNA level, was associated with sensitivity to initial chemotherapy and the survival of patients with AML, especially AML-M5 (Yokoyama et al, 1996). Here we have confirmed that AML-M5 patients (six M5 cases) with low *nm23-H1* expression levels under 100 exhibited significantly higher rates of survival than those (eight M5 cases) with higher levels over 100 (log-rank test, $P < 0.025$). We also found that elevated *nm23-H1* mRNA levels were associated with significantly reduced survival of AML-M2 patients (high eight M2 cases vs low 11 M2 cases), while the presence of t(8;21) in AML-M2 patients was not associated with the overall survival (Figure 2). It is widely accepted that patients with t(8;21) have a better remission rate than the mean for other subjects, but there was no statistical difference in overall survival in our study. A similar result was not obtained in AML-M1 patients (high five M1 cases vs low nine M1 cases, log-rank test, $P < 0.99$). We could not analyse the AML-M3 and -M4 cases because there were few or no cases with *nm23-H1* expression levels higher than 100. Thus, these results indicate that the *nm23-H1* mRNA level is a prognostic factor not only for AML but also for AML-M2 and -M5 subtypes.

We observed a strong correlation between *nm23-H1* and *-H2* expression levels in AML ($r = 0.726$, Figure 1), although we have previously reported that *nm23-H1* but not *-H2* was a prognostic factor for AML. Therefore we analysed the involvement of the *nm23-H2* mRNA expression level in the chemotherapy sensitivity

© Cancer Research Campaign 1998

and survival probability of patients. As shown in Table 4, the CR ratios of groups A, B, C and D were 50%, 73%, 57% and 100% respectively. Although the groups with higher expression levels of *nm23-H1* (group A + C) showed lower CR ratios than did the other groups, the CR ratio of group A was similar to that of group C. The CR ratios of the groups with higher expression levels of *nm23-H2* (group A + D) were similar to those of the other groups. The sensitivity to initial chemotherapy seems to be associated with the *nm23-H1* expression level rather than the *nm23-H2* expression level.

Next, we compared the survival probability between the four groups; group A vs B, A vs C, A vs D, B vs C, B vs D and C vs D. Group B exhibited significantly longer survival times compared with group A (log-rank test, $P < 0.005$, Figure 3A and Table 5), although the comparisons between all the other groups indicated no significant difference in survival time (Table 5). When we compared the survival probability between the two groups divided by *nm23-H1* expression levels (group A+C vs group B+D), group B+D exhibited significantly longer survival times than did group A+C (log-rank test, $P < 0.01$, Table 5). The survival probability between the two groups divided by *nm23-H2* expression levels (group A+D vs group B+C) seems to be different, but the difference was not statistically significant (log-rank test, $0.05 < P < 0.10$, Table 5). These results indicate that lower expression levels of both *nm23-H1* and *-H2* is a better prognostic factor than that of only *nm23-H1*, as the *P*-value obtained by the log-rank test of A vs B is higher than that of A+C vs B+D. As described above, most AML-M3 cases belong to group B, and this group exhibited significantly longer survival times than did group A (log-rank test $P < 0.005$, Figure 3A). AML-M3 cases in group B (group B1) exhibited significantly longer survival times in comparison with non-M3 cases in the same group (group B2, log-rank test, $P < 0.05$). The survival times of group B2 are statistically longer than those of group A (log-rank test, $P < 0.05$, Figure 3B), suggesting that lower expression levels of both *nm23-H1* and *-H2* are associated with good prognosis in AML. In the AML-M3 cases, there may be other factors regulating the abnormal growth and differentiation of the leukaemic cells.

### DISCUSSION

*nm23-H1* was discovered on the basis of its reduced expression level in highly metastatic murine melanoma cell lines, compared with related, weakly metastatic melanoma cell lines (Steeg et al, 1988). *nm23-H2*, a closely related gene, was identified by cross-hybridization with *nm23-H1* on screening of a human fibroblast cDNA library (Stahl et al, 1991). Reduced *nm23-H1* expression levels have been correlated with reduced patient disease-free or overall survival time or other histopathological indicators of high metastatic potential in cohorts of breast, ovarian, cervical, gastric and hepatocellular carcinoma and melanoma. However, the opposite trend has been identified in neuroblastoma, pancreatic carcinoma, lymphoma and leukemia (De La Rosa, 1995). We previously reported that *nm23-H1* and *nm23-H2* were overexpressed in AML and the higher significance of *nm23-H1* expression correlated with a poor prognosis in AML. It has also been reported that among malignant lymphomas, high-grade non-Hodgkin's lymphoma (NHL) and Hodgkin's lymphoma samples exhibited significantly higher *nm23-H1* expression levels than did low-grade NHL samples. These studies suggest that *nm23-H1* expression in human haematopoietic malignancies is associated with disease aggressiveness. In this study, we confirmed the clinical implication of *nm23-H1* expression for AML on a larger scale than in our previous study and clarified the involvement of *nm23-H2* expression levels on the prognosis of AML, as *nm23-H2* was also significantly overexpressed in AML (Table 1), and a statistically significant correlation between the levels of expression of *nm23-H1* and *-H2* was observed in AML (Figure 1). As reported previously for the 42 AML cases, the overall survival of the AML patients with a high (>100) or low (<100) *nm23-H2* index was not statistically different ($P = 0.6384$), even when the cut-off points were varied. In this study of 76 AML cases, the overall survival of the AML patients with a low *nm23-H2* index (group B+C) was not significantly higher than those with a high *nm23-H2* index (group A+D), although the *P*-value came close to being significant ($0.05 < P < 0.10$) (Table 5). Group B (both expression levels low) exhibited significantly longer survival times than did group A (both expression levels high) (log-rank test, $P < 0.005$, Figure 3A and Table 5). These results suggest that the *nm23-H2* expression level may be a weak prognostic factor for AML and the combination of *nm23-H2* and *nm23-H1* expression is a better prognostic factor than only *nm23-H1* expression alone.

Based on analysis of the promoter regions of *nm23-H1* and *-H2*, it is suggested that both *nm23* genes are independently and differentially regulated (Okada et al, 1996). However, a significant correlation between the levels of expression of *nm23-H1* and *-H2* was observed in AML (Figure 1). Interestingly, group D seemed to have a good response to the first chemotherapy and 50% of group D were AML-M4 cases (Table 4). The ratio of *nm23-H1* to *nm23-H2* expression levels may provide useful information, as the ratio (H1/H2) in total AML was higher than that for normal blood cells and similar to that for the HEL leukaemic cell line, and the ratio in AML-M3 and -M4 cases is close to that of normal cells (Table 1).

In this study, we also clarified the correlation of *nm23* expression levels with FAB classification (Table 1 and Table 3). Among AML subtypes, AML-M5 showed relatively higher expression levels than did the other FAB subtypes, although the difference in *nm23* expression levels between M5 and non-M5 AML was not statistically significant (Table 3). On the other hand, we have found that AML-M3 cases have the lowest *nm23* expression levels, a lower H1/H2 ratio and better prognosis in comparison with the other FAB subtypes in AML. The low expression of *nm23* in AML-M3 may be associated with granulocytic differentiation-associated characteristics and with potential to differentiate into mature cells with all-*trans* retinoic acid (ATRA). nm23 proteins were purified as differentiation inhibitory factors from non-differentiating myeloid leukaemia cells (Okabe-Kado et al, 1992). Yamashiro et al (1994) have reported that down-regulation of *nm23* gene expression is observed during the induced differentiation of some human leukaemia cell lines. These results suggest that AML-M3 cells are at a certain stage of granulocytic maturation and down-regulation of *nm23* gene expression accompanies the maturation. It remains to be determined whether inhibition of *nm23* expression affects the induction of the differentiation of leukaemia cells.

AML-M6 (erythroleukaemia) cases had extremely high expression levels of both *nm23* genes and the ratio (H1/H2) was also extremely high, although only two cases were investigated (Table 1). It would be interesting to clarify whether the erythroid differentiation-associated properties are related to the overexpression of *nm23* genes.

© Cancer Research Campaign 1998

Recently the nm23 protein was reported to have a specific interaction with ROR/RZR nuclear receptors (Paravicini et al, 1996), and the interaction required the presence of the N-terminal 60 amino acids of the nm23 protein predicted to be important for the I-factor activity of nm23 protein (Okabe-Kado et al, 1995). The receptors for the retinoids are prominent members of the nuclear receptor superfamily and ROR/RZR receptors have been identified by homology cloning. They are known as orphan receptors, referring to the fact that their ligands are unknown. RORα is expressed in a variety of organs with the highest levels of specific mRNA detected in leucocytes. It is of interest to examine the expression levels of RORα in leukaemia cells. Although the biological implication of ROR/RZR is unknown, it is tempting to speculate that retinoic acid receptor (RAR) and/or other nuclear receptors might interact with the nm23 protein and participate in the down-regulation of *nm23* expression. A good prognosis in AML-M3 is not solely related to *nm23* expression, as the survival times of AML-M3 cases were much longer than those of non-M3 AML cases in the same low *nm23* expression level group (Figure 3). AML-M3 is associated with a consistent t(15;17) translocation that fuses the promyelocytic leukaemia (PML) gene to the RARα gene. Although the PML-RAR fusion protein is a major prognostic factor in AML-M3, the translocation (15;17)(q22;q12) found in AML-M3 might affect the expression of *nm23* genes located on chromosome 17q21. We are now examining whether the levels of *nm23* expression affect the sensitivity of AML-M3 patients to ATRA treatment and whether they change during the disease progression of AML-M3 patients.

### ACKNOWLEDGEMENTS

This work was supported in part by a grant from the Ministry of Health and Welfare, and Grants-in-Aid for Scientific Research (C) and Cancer Research, from The Ministry of Education, Science, Sports and Culture, Japan, and a grant from the Kawano Memorial Foundation for Promotion of Pediatrics.

### REFERENCES

AML-87 Study of the Japan Adult Leukemia Study Group (1993) Randomized study of individualized induction therapy with or without vincristine, and of maintenance–intensification therapy between 4 or 12 courses in adult acute myeloid leukemia. *Cancer* **71**: 3888–3895

Backer JM, Mendra CE, Kovesdi I, Fairhurst JL, O'Hara B, Eddy RL, Shows TB, Methew S, Murty VVVS and Chaganti RSK (1993) Chromosomal localization and nucleoside diphosphate kinase activity of human metastasis-suppressor genes *NM23-1* and *NM23-2*. *Oncogene* **8**: 497–502

Chandrasekharappa SC, Gross LA, King SE and Collins FS (1993) The human *NME2* gene lies within 18 kb of *NME1* in chromosome 17. *Genes Chrom Cancer* **6**: 245–248

Chomczynski P and Sacchi N (1987) Single-step method of RNA isolation by guanidium thiocyanate–phenol–chloroform extraction. *Anal Biochem* **162**: 156–159

De La Rosa A, Williams RL and Steeg PS (1995) *Nm23*/nucleoside diphosphate kinase: toward a structural and biochemical understanding of its biological functions. *BioEssays* **17**: 53–62

Gilles AM, Presecan E, Vonica A and Lascu I (1991) Nucleoside diphosphate kinase from human erythrocytes. *J Biol Chem* **266**: 8784–8789

Ohno R, Ohnishi K, Takeshita A, Tanimoto M, Murakami H, Kanamoto A, Norio A, Kobayashi T, Kuriyama K, Ohmoto E, Sakamaki H, Tsubaki K, Hiraoka A, Yamada O, Oh H, Furusawa S, Matsuda S, Naoe T (1994) All-*trans* retinoic acid therapy in relapsed/refactory or newly diagnosed acute promyelocytic leukemia (APL) in Japan. *Leukemia* **8**(suppl. 3): 64–69

Okabe-Kado J, Kasukabe T, Honma Y, Hayashi M, Henzel WJ and Hozumi M (1992) Identity of a differentiation inhibiting factor for mouse myeloid leukemia cells with nm23/nucleoside diphosphate kinase. *Biochem Biophys Res Commun* **182**: 987–994

Okabe-Kado J, Kasukabe T, Hozumi M, Honma Y, Kimura N, Baba H, Urano T and Shiku H (1995*a*) A new function of *Nm23*/NDP kinase as a differentiation inhibitory factor which does not require its kinase activity. *FEBS Lett* **363**: 311–315

Okabe-Kado J, Kasukabe T, Baba H, Urano T, Shiku H and Honma Y (1995*b*) Inhibitory action of *nm23* proteins on induction of erythroid differentiation of human leukemia cells. *Biochim Biophys Acta* **1267**: 101–106

Okada K, Urano T, Goi T, Baba H, Yamaguchi A, Furukawa K and Shiku H (1994) Isolation of human *nm23* genomes and analysis of loss of heterozygosity in primary colorectal carcinomas using a specific genomic probe. *Cancer Res* **54**: 3979–3982

Okada K, Urano T, Baba H, Furukawa K and Shiku H (1996) Independent and differential expression of two isotypes of the promoter regions of the nm23-H1 and H2 genes. *Oncogene* **13**: 1937–1943

Paravicini G, Steinmayr M, Andre E and Becker-Andre M (1996) The metastasis suppressor candidate nucleoside diphosphate kinase *nm23* specifically interacts with members of the ROR/RZR nuclear orphan receptor subfamily. *Biochem Biophys Res Commun* **227**: 82–87

Stahl JA, Leone A, Rosengard AM, Porter L, King CR and Steeg PS (1991) Identification of a second human *nm23* gene, *nm23-H2*. *Cancer Res* **51**: 445–449

Steeg PS, Bevilacqua G, Kopper L, Thorgeirsson UP, Talmadge JE, Liotta LA and Sobel ME (1988) Evidence for a novel gene associated with low tumor metastatic potential. *J Natl Cancer Inst* **80**: 200–204

Yamashiro S, Urano T, Shiku H and Furukawa K (1994) Alteration of *nm23* gene expression during the induced differentiation of human leukemia cell lines. *Oncogene* **9**: 2461–2468

Yokoyama A, Okabe-Kado J, Sakashita A, Maseki N, Kaneko Y, Hino K, Tomoyasu S, Tsuruoka N, Kasukabe T and Honma Y (1996) Differentiation inhibitory factor *nm23* as a new prognostic factor in acute monocytic leukemia. *Blood* **88**: 3555–3561

© Cancer Research Campaign 1998